# Does ant–plant mutualism have spillover effects on the non‐partner ant community?

**DOI:** 10.1002/ece3.8524

**Published:** 2022-01-24

**Authors:** Marion L. Donald, Tom E. X. Miller

**Affiliations:** ^1^ Program in Ecology and Evolutionary Biology Department of BioSciences Rice University Houston Texas USA; ^2^ Biocontrol & Molecular Ecology Manaaki Whenua Landcare Research Lincoln New Zealand

**Keywords:** Ant–plant, composition, diversity, mutualism, positive feedback

## Abstract

Mutualism benefits partner species, and theory predicts these partnerships can affect the abundance, diversity, and composition of partner and non‐partner species. We used 16 years of monitoring data to determine the ant partner species of tree cholla cacti (*Cylindropuntia imbricata*), which reward ants with extrafloral nectar in exchange for anti‐herbivore defense. These long‐term data revealed one dominant ant partner (*Liometopum apiculatum*) and two less common partners (*Crematogaster opuntiae* and *Forelius pruinosus*). We then used short‐term characterization of the terrestrial ant community by pitfall trapping to sample partner and non‐partner ant species across ten plots of varying cactus density. We found that the dominant ant partner tended a higher proportion cacti in plots of higher cactus density, and was also found at higher occurrence within the pitfall traps in higher density plots, suggesting a strong positive feedback that promotes ant partner occurrence where plant partners are available. Despite the strong association and increased partner occurrence, ant community‐wide effects from this mutualism appear limited. Of the common ant species, the occurrence of a single non‐partner ant species was negatively associated with cactus density and with the increased presence of *L. apiculatum*. Additionally, the composition and diversity of the ant community in our plots were insensitive to cactus density variation, indicating that positive effects of the mutualism on the dominant ant partner did not have cascading impacts on the ant community. This study provides novel evidence that exclusive mutualisms, even those with a strong positive feedback, may be limited in the scope of their community‐level effects.

## Graphical abstract

Theory predicts that community composition and diversity should be affected by mutualist promotion of partner relative abundance and shifts in a competitive hierarchy. Despite these clear predictions and evidence of promotion of the dominant mutualist ant partner, we did not find strong spillover effects on the ant community composition or diversity.

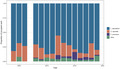

## 1 INTRODUCTION

Mutualisms, classically defined as positive interactions between species, are increasingly recognized as forces that can structure communities (Bronstein et al., 2006; Stachowicz, 2001; Traveset & Richardson, 2014). Mutualists are embedded within larger communities of other partner and non‐partner species with which they interact as mutualists, competitors, predators, or prey (Aranda‐Rickert et al., 2014; Blüthgen et al., 2004; Palmer et al., 2003). Theory predicts that the positive benefits between two species can spill over to influence community composition and diversity by changing the relative abundance and competitive hierarchies. These interactions may function as the opposite of keystone predation, in which predators promote diversity through the suppression of competitively dominant species (Paine, 1966). For instance, mutualisms commonly drive positive feedback between partners that can promote partner species’ occurrence and abundance with resulting decreases in non‐partner species’ abundance and community diversity (Frederickson et al., 2005; Rudgers & Clay, 2008; Rudgers et al., 2010; Styrsky & Eubanks, 2007). However, the outcomes of mutualistic interactions on the larger community should depend on whether the benefiting partner species are superior or inferior competitors and the degree of niche overlap between partner and non‐partner species. Given the ubiquity of mutualisms in nature, understanding whether and how mutualisms affect partner and non‐partner species and community diversity is an important and growing line of inquiry.

Ant–plant interactions involving an exchange of food for protection are ideal model systems for understanding the community effects of mutualism. Globally, 332 genera of plants are known to produce extrafloral nectar (EFN) (Koptur, 1992), which can serve as a dietary resource for numerous ant species (Baker et al., 1978; Davidson, 1997). EFN has been shown to promote ant abundance and colony size (Byk & Del‐Claro, 2011), and this dietary resource is often exchanged for deterrence of herbivores (Chamberlain & Nathaniel Holland, 2009; Rosumek et al., 2009; Trager et al., 2010). Despite the potential importance of EFN for ants, the majority of ant–plant systems are studied unilaterally from the plant's perspective (Bronstein, 1994; Lanan & Bronstein, 2013). There is strong evidence for ant partners reducing herbivory (Rudgers et al., 2010) and herbivore diversity (Pringle & Gordon, 2013), and enhancing nutrient availability (Mayer et al., 2014; Wagner & Fleur Nicklen, 2010), growth, and survival (Báez et al., 2016), all of which benefit the plant partners. Much less is known about the consequences of ant–plant mutualism from the ant's perspective (but see Byk & Del‐Claro, 2011 and Lanan & Bronstein, 2013). Focus on the effects of plants on ants would help document the complete feedback loop between mutualist partners.

While some well‐studied ant–plant mutualisms are obligate for the ants (e.g., the ant symbionts of myrmecophytic *Acacia* spp.), many other common yet less well‐studied ant–plant mutualisms are facultative. Facultative mutualisms can be diffuse, with multiple ant species associating with one or more plant partner species (Alonso, 1998; Horvitz & Schemske, 1990). Plants engaged in facultative mutualisms with ants often provide some dietary resources but not housing, and thus, the ants must rely on other resources to meet their requirements. This dependence on non‐plant resources likely thrusts facultative ant partners into competition with non‐partner ant species for nest space and nitrogen‐rich dietary resources. Indeed, even for ant species with little dietary overlap, competition for nesting sites can be intense (Levings & Franks, 1982). Given that ant communities are often strongly structured by competition (Hölldobler et al., 1990; Styrsky & Eubanks, 2007; Toby Kiers et al., 2010), if EFN promotes the occurrence and abundance of partner ant species, non‐partner ant species may be negatively affected, with consequences for community richness, evenness, and composition.

In this study, we addressed the central hypothesis that facultative mutualism between ants and EFN‐bearing plants has cascading effects on the broader ant community, including partner and non‐partner species. This hypothesis makes two predictions for associations between ants and cacti that our observational study addressed. First, if ant defenders and EFN‐bearing plants engage in positive feedback, then partner ant species should be more abundant, on and off plants, in areas where EFN resources are more abundant. Second, if increased abundance of partner ant species escalates competition for nest sites and other non‐EFN resources with non‐partner species, then areas of high EFN and ant partner abundance should see declines in non‐partner abundance, reductions in community diversity, and/or shifts in composition. Our observational approach relied on long‐term data to identify key ant partner species and short‐term spatial sampling to characterize ant communities spanning a natural gradient of EFN–plant densities.

Our study focused on the EFN‐bearing cactus *Cylindropuntia* (=*Opuntia*) *imbricata* Haw. [D.C.] and its ant partners in the northern Chihuahuan Desert. Previous work in this system based on short‐term observations and experiments identified benefits between one of the ant partner species and the cactus (Miller, 2007; Ohm & Miller, 2014). Based on this, we anticipated that if there was long‐term consistency in ant partner identity, this would likely result in the promotion of partner abundance elsewhere in the environment (off of the cacti). We used 16 years of plant census data to identify the dominant ant partner species and the temporal consistency of ant–plant associations. Then, in a single year, we surveyed ant communities in plots of varying cactus density. The observational surveys characterized ant communities independently of the ant–cactus mutualism (using pitfall traps). These two types of information allowed us to test whether on‐plant associations with alternative partner species reflect their abundance in the environment, or whether dominant partners are over‐represented on plants relative to their occurrence in the environment. As the cacti are likely longer‐lived (>40 years) and less mobile than the ant colonies, and the benefits of ant tending have been previously described (Miller, 2007; Ohm & Miller, 2014), we framed our hypotheses around the response of ant partner species presence and abundance to cactus density, and the resulting effects on the ant community. We used the long‐term data and short‐term ant community characterization to answer the following specific questions:

1. Which ant species is/are the dominant partner(s) and how consistent are these associations?
2. What is the association between partner and non‐partner ant species with spatial variation in cactus density, a proxy for EFN resource availability?
3. Does the frequency of alternative ant partners on cacti reflect their occurrence or abundance in the environment?
4. What are the spillover effects of the ant–plant mutualism on ant community diversity and composition?

## 2 METHODS

### 2.1 Natural history and study area

The tree cholla (*C. imbricata*) is a long‐lived cactus common throughout the Chihuahuan Desert and native to arid grasslands across the southwestern United States. Its growth form is arborescent with cylindrical, photosynthetic stems and large, conspicuous spines (Benson, 1982; Fraser & Pieper, 1972). Tree cholla cacti secrete EFN from specialized glands on young vegetative and reproductive structures (nectaries are on the outside of flower buds and fruits), as in other EFN‐producing cactus species (Ness et al., 2016; Oliveira et al., 1999). Previous work at our study site found that reproductive structures produce greater amounts of EFN than do vegetative structures (Miller, 2014). Tree cholla secrete EFN throughout the growing season, April through September in our study region.

We conducted this study at the Sevilleta National Wildlife Refuge, a Long‐term Ecological Research (LTER) site in Socorro County of central New Mexico (34°20′5.3″N, 106°37′53.2″W). Our sampling sites were located on the west‐facing slopes of the Los Piños Mountains (1790 m). The habitat includes rocky soils and perennial vegetation characteristic of the high Chihuahuan Desert, including grasses (*Bouteloua gracilis* and *B. eriopoda*), other cacti (*Opuntia* and *Echinocereus*), *Yucca*, and oaks (*Quercus*). In this habitat, tree cholla cacti are the primary source of nectar (co‐occurring cactus species do not produce EFN [T.E.X. Miller, Pers. Obs.]), though we have observed occasional ant‐tending of aphids on oaks. As *C. imbricata* is the only source of EFN, this characteristic of the system allowed us to address how EFN availability is associated with ant partner identity and ant community composition and diversity.

Three ant species are known from previous studies to collect tree cholla EFN in our study area (Miller, 2007, 2014; Ohm & Miller, 2014): *Crematogaster opuntiae* Buren, *Liometopum apiculatum* Mayr, and *Forelius pruinosus* Roger. All of these species are ground‐nesting and differ in their interaction outcomes with tree cholla cacti. *L. apiculatum* is an effective bodyguard against cactus herbivores and seed predators, while *C. opuntiae* is less so and may even have a net parasitic effect by deterring pollinators (Miller, 2007; Ohm & Miller, 2014). The effects of *F. pruinosus* on tree cholla fitness are not known. Additionally, ants in the genera *Liometopum* and *Forelius* are considered competitively dominant, while *Crematogaster* spp. tend to be less so (Andersen, 1997). Further, *L. apiculatum* often relies on carbohydrate‐rich exudates (de Conconi et al., 1983; Corona et al., 2007) as a key part of this ant species’ diet. When tree cholla individuals are tended, it is almost always by a single ant species at a given time. However, closely co‐occurring cacti may be tended by different ant species. And short‐term studies (Miller, 2007, 2014) show that ant occupant identity on individual cacti tends to remain constant within and across years, with changes typically being unidirectional from *C. opuntiae* to *L. apiculatum*.

### 2.2 Data collection

#### 2.2.1 Long‐term monitoring

To document which ant species are the most frequent partners on plants through time, and therefore most likely to engage in positive feedback with plants, we surveyed ant presence and identity on marked cacti from 2004 to 2019 (no survey data were collected during 2007). For the years 2004–2008, the survey included a total of 127 plants distributed across three spatial blocks (ca. 40 cacti / block), separated by ca. 2 km. In 2009, we stopped this census and began a new census in six 30 × 30 m plots that were separated by 0.5–2 km. In each year (2009–2019), we visited all cacti (mean: 87 cacti / plot) and assessed each for ant partner identity and recorded whether the cacti were reproductive or vegetative. Two additional plots of the same dimensions were added in 2011 and censused each subsequent year as above. Finally, in 2013 two additional plots were included solely in that year's census and ant community measurements (described below), for a total of ten plots in that year. In total, we made 8298 observations of 1225 individual cacti between 2009 and 2019. Cacti were surveyed a minimum of one year and a maximum of 11 years (mean ± SD: 6.5 ± 3.6 years). During these surveys, reproductive status, survival, ant occupancy, and other variables described in Miller (2007, 2014) were recorded. These surveys occurred in late May or early June of each year.

#### 2.2.2 Cactus density and vegetation cover

Because our study relied on natural variation in cactus density (as a proxy for EFN resources), it is possible that overall cactus density may not be correlated with reproductive cactus density (the dominant source of EFN) and/or is confounded with other habitat variables. To evaluate this, we counted the number of reproductive cacti within each plot, and quantified vegetation cover and substrate type in each plot. We identified the percent cover of vegetation (grass, shrub, woody plant (oaks and junipers), forb, cactus, and other (e.g., *Nolina* sp.)) and substrate (vegetated, bare soil, and rock) in eight 1 × 1 m quadrats that were located in a standardized grid within each plot on May 29, 2013.

#### 2.2.3 Ant community sampling

We used pitfall traps to sample the ant communities in each of the 10 plots in May 2013. This method allowed us to sample partner and non‐partner ant species independently of the mutualism. In each plot, we arranged 16 pitfall traps (cylindrical plastic containers 5 cm in diameter) in a 4 × 4 standardized grid, inset 3 m from the plot boundaries and 8 m apart from one another. We placed the pitfall traps in the ground so that the rim was flushed with the ground surface. We filled the pitfall traps with water mixed with several drops of unscented soap to break the surface tension. We set the pitfall traps out for a 24‐h period from 10 a.m. on May 29, 2013, to 10 a.m. on May 30, 2013. Based on weather data from the nearby Blue Grama Meteorological Station of the Sevilleta LTER, the mean temperature during this 24‐h period was 22.37°C and there was no rainfall.

We used MacKay and Mackay (2002) and Fisher and Cover (2007) to identify ant specimens to genus and, when possible, species. Pitfall traps yielded data on ant species occurrence (presence or absence in a trap) and abundance (number of ants in a trap, if present). We recorded both of these metrics and use them in our analyses below. However, we interpret our abundance data cautiously, as previous works by Romero and Jaffe (1989) and Andersen (1991) have found that the abundance data for ants from pitfall traps may not accurately represent the actual abundance of a species, due to biases by worker trails and different locomotive behaviors.

### 2.3 Statistical analyses

#### 2.3.1 Long‐term ant partner identities

For each year of the long‐term data, we calculated the proportion of cacti tended by each ant species (grouping unknown and very uncommon ant partners that were observed <1% of the time as “other”), and visualized the frequencies of association through time. We excluded cacti that were not tended (lacking any ants) from this analysis because these are generally very small plants that do not produce EFN (Miller, 2014) and are therefore effectively unavailable as mutualist partners.

#### 2.3.2 Cactus density and ground cover composition

To assess whether overall cactus density (reproductive and non‐reproductive cacti) reflects the density of reproductive cacti in each plot in 2013, we used linear models with a Gaussian distribution. We first fit a null model, and then a model with the fixed effect of cactus density as a predictor variable and reproductive cactus density as the response variable, and used an F test to determine whether adding the predictor variable of cactus density significantly improved the fit to the data. As this initial analysis found overall cactus density to be predictive of reproductive cactus density, we then proceeded with using overall cactus density as our predictor variable in subsequent analyses.

To assess whether cactus density across the plots was correlated with ground cover composition in the plots, we transformed our percent cover matrix to a Bray–Curtis dissimilarity matrix and transformed cactus density into a Euclidean distance matrix with the R package “ecodist” (Goslee & Urban, 2007). We then conducted a Mantel test to determine the correlation between these matrices. These and all other analyses were performed in R v.3.6.3 (R Core Team, 2020).

#### 2.3.3 Association of ant partners on cacti with cactus density

For each of the ant partner species identified from the long‐term data, we used generalized linear mixed‐effects models to ask whether the fraction of plants that they tended in a plot was related to cactus density. We fit a Bernoulli response distribution of ant occurrence (present/absent) on a cactus. The fixed effect was cactus density, and the random effect was plot identity to reflect background plot‐to‐plot variance. Each of these models was compared with a null model with the response variable of ant occurrence and the random effect of plot. We calculated likelihood ratios of these nested models and used chi‐squared tests to determine whether adding the predictor variable of cactus density significantly improved the fit to the data. All models were fit with package lme4 (Bates et al., 2015), and fit was assessed using DHARMa (Hartig, 2021).

#### 2.3.4 Association of common ant species in pitfalls with cactus density and with the dominant ant partner

For the abundance and occurrence analyses from the pitfall traps, we restricted our focus to the four most common ant species. This was because all other ant species occurred infrequently in the traps (<15% of all traps), except for *Camponotus* sp. 2, which occurred in approximately 17% of all traps but at a very low abundance (0.244 +/‐ 0.048 workers, mean +/‐ SE). The four common species included the three partner and one non‐partner ant species, allowing us to contrast the results across ant species that differ in the strength of their association with the cactus.

We used a hurdle approach for our zero‐inflated ant count data, where we fit two separate generalized mixed‐effects models to ask whether the occurrence and abundance (conditional on occurrence) of these common ant species within pitfall traps responded to variation in cactus density. The fixed effect was cactus density, and the random effect was plot identity. Each of these models was compared to a null model with the response variable of ant occurrence and the random effect of plot. This was done using a chi‐squared test to determine the importance of cactus density as a predictor of either ant occurrence or abundance. Occurrence models used a Bernoulli response distribution, and abundance models used a Gaussian distribution with abundance being natural log‐transformed.

Additionally, as *L. apiculatum* was observed to be the dominant ant partner through time, we also fit occurrence and abundance models as described above for the three common ant species in response to the occurrence and abundance of *L. apiculatum*, respectively. As before, these models included plot identity as a random effect, and we use the same methods of model comparison to determine the importance of *L. apiculatum* as a predictor for the occurrence and abundance of the other ant species.

#### 2.3.5 Association of ant community diversity and composition with cactus density

To determine whether positive feedback between cactus and ant partner abundance affected ant community diversity and composition, we examined community species richness, Shannon diversity (a measure of community evenness), and composition. We used the R package “vegan” (Oksanen et al., 2019) to calculate Shannon diversity and used generalized linear mixed models to evaluate the relationship between cactus density and ant community richness and evenness. These models were fit with a Gaussian distribution, including plot as a random effect, and were assessed for fit using the methods described above. Additionally, richness data were log‐transformed, and the one pitfall trap in which zero ants were collected was dropped from these community analyses.

Finally, we assessed how ant community composition varied with cactus density. We used PCoA to visualize the ant communities captured in the pitfall traps using both Jaccard (presence/absence) and Bray–Curtis dissimilarity matrices. Abundance data were log‐transformed for the Bray–Curtis dissimilarity matrix. We then used PERMANOVA in the vegan package to test whether ant community composition was related to cactus density. To account for the repeated sampling of the pitfall communities within each plot, we set “strata” equal to plot identity within the “adonis” function in the “vegan” package. Additionally, to ensure that the fine resolution of the pitfall‐level data (which can be strongly affected by patchiness of ant foraging and nesting) was not obscuring broader patterns, we repeated the above community analyses with all of the pitfall data pooled within plot. For these analyses, we ran linear models and used ANOVA to assess fit, and we used PERMANOVA without strata set to plot.

## 3 RESULTS

### 3.1 Long‐term ant partner identities

**FIGURE 1:**
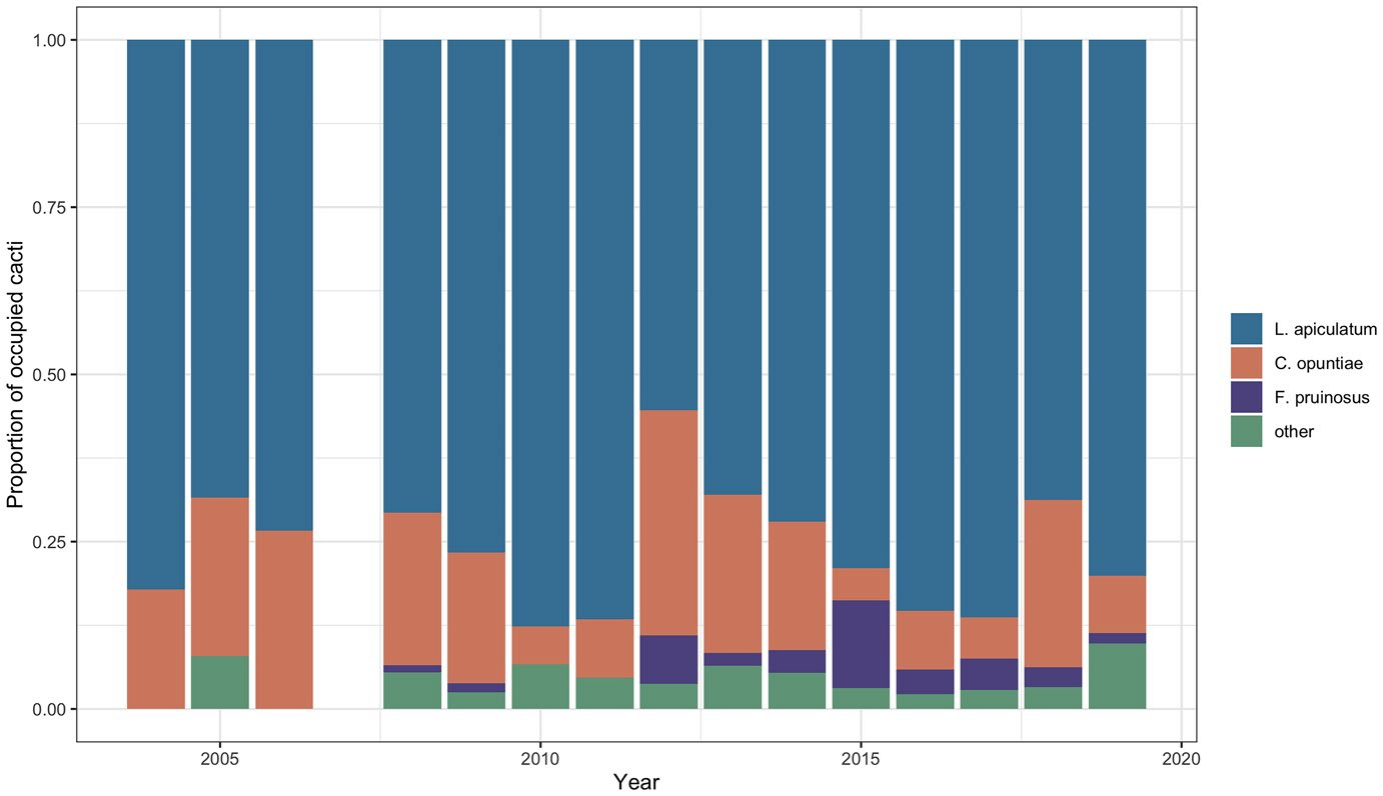
Proportion of tended tree cholla cacti by *Liometopum apiculatum* (blue), *Crematogaster opuntiae* (orange), *Forelius pruinosus* (purple), and other ant species (green) across the 16‐year‐long monitoring study. Note that no ant survey data were collected in 2007

Data across 16 years of monitoring revealed that *L. apiculatum* was the dominant ant partner species of tree cholla cacti. Across all of the years surveyed, 75.6% of tended cacti, on average, were tended by *L. apiculatum* (Figure 1). *Crematogaster opuntiae* was the second most frequent partner, occupying 16.4% of the total tended cacti, on average, while *F. pruinosus* and all other ant species were infrequent partners (<4.5%) (Figure 1). These rankings of partnerships were highly stable throughout the 16‐year period. There was only one year (2015), where *F. pruinosus* was a more frequent partner than was *C. opuntiae*. Of the total cacti present, 25.2% to 80.7% (mean ± SE: 55.7% ± 4.0%) hosted ant partners across the study years. This variation in percentage of occupied cacti may be due to numerous factors across the years including EFN production in response to weather and temporal variation in ant reliance on EFN.

### 3.2 Ant community characterization

Of the 160 pitfall traps placed in the 10 plots in 2013, 159 were recovered. We identified 10 genera and a total of 13 species or morphospecies (Table S1). Out of 159 pitfalls containing 7637 ant specimens, two species (*Pheidole* sp. 1 and *Forelius pruinosus*) were found in >60% of all traps and accounted for ca. 68% of all ants captured. The partner species *L. apiculatum* and *C. opuntiae* were found in ca. 20% and 16% of all traps, respectively. These four species were the most abundant ant species in our pitfall traps, collectively making up 95.46% of all ants captured. The other nine species each occurred in less than 13% of the traps, except for *Camponotus* sp. 2, which occurred in 16.98% of the traps but at a very low mean abundance (0.24 ± 0.048 ants).

### 3.3 Cactus density and ground cover composition

Reproductive cactus density was positively associated with overall cactus density (*F* = 6.7214, *df* = 1, *p* = .03199, adjusted *R* ^2^ = .39), indicating that overall cactus density captures the contributions of reproductive cacti and thus serves as a good proxy for EFN resources. Overall, cactus density also accounts for the non‐reproductive cacti, which produce EFN in lesser amounts.

Tree cholla cactus density doubled across our 10 study plots, from 0.05 to 0.11 plants m^−2^. Ground cover was otherwise similar across plots, with consistently high cover of bare ground, rock, and grass, and not significantly correlated with the variation of cactus density (Figure S1; Mantel’s test statistic *r* = −.0159, *p* = .5193 with 10,000 permutations).

### 3.4 Association of ant partners on cacti with cactus density

**FIGURE 2:**
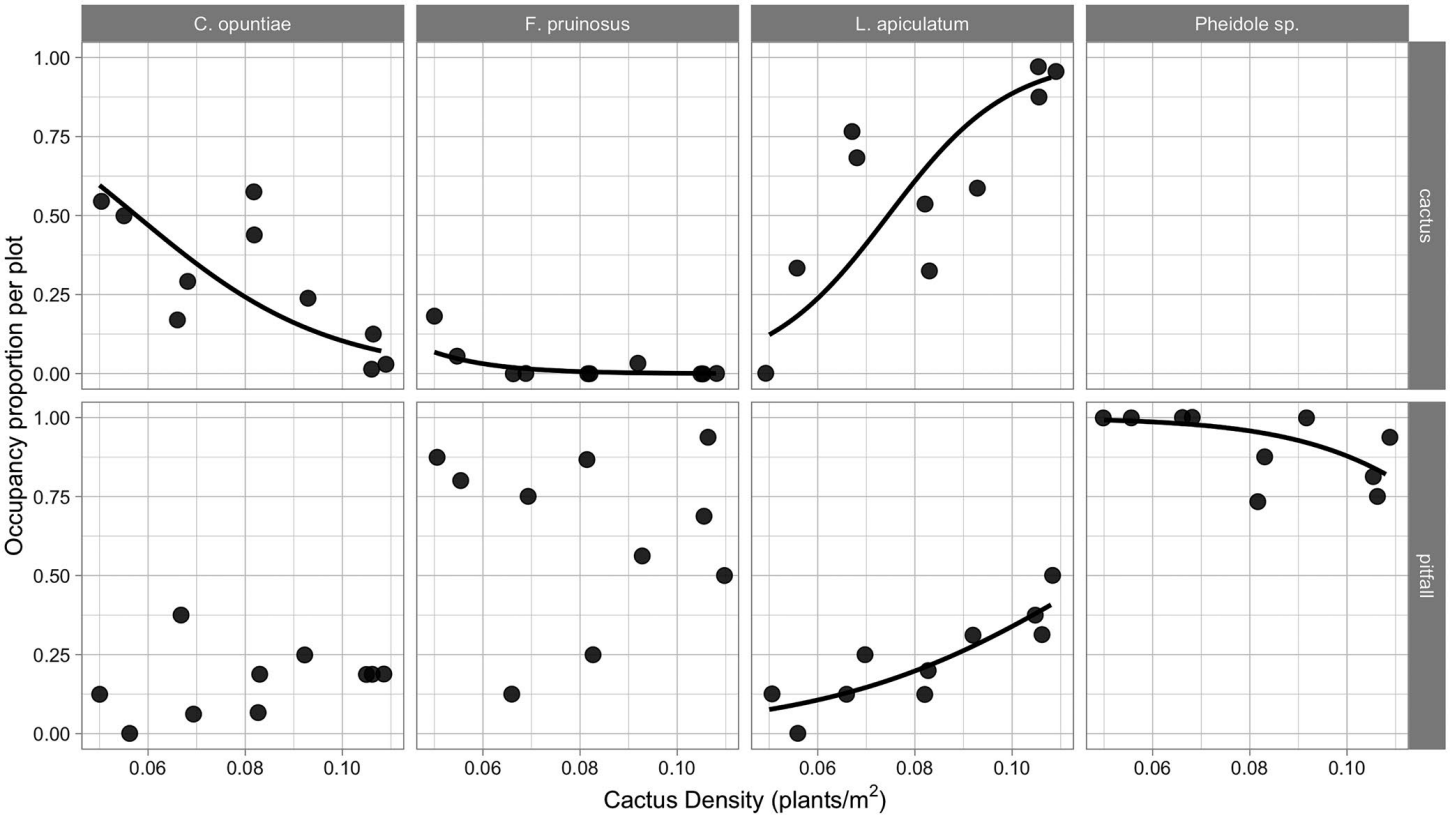
Occupancy proportion for each of the four most common ant species per plot across the natural gradient of cactus density. Responses of the four most common ant species are shown in columns, and occupancy on cacti or in pitfall traps is shown in rows. Each point represents the proportion of either cacti tended or pitfall traps occupied by the ant species denoted by each row in relation to the cactus density of the plot. Note that *Pheidole* sp. was not observed on cacti, and therefore, the cactus panel is blank. Lines represent significant model fits of occupancy responding to cactus density

We found that the occurrence of *L. apiculatum* on individual cacti was positively associated with cactus density at the plot level (Figure 2; χ^2^ = 10.45, *df* = 1, *p* = .00122), suggesting that this species gains access to a greater fraction of cacti in areas where they are more abundant. Specifically, the probability of occurrence of *L. apiculatum* on cacti increased from 13% to 94% across the gradient of cactus density. In contrast, occurrences of *C. opuntiae* and *F. pruinosus* on individual cacti were negatively associated with cactus density (Figure 2; *C. opuntiae*: χ^2^ = 8.319, *df* = 1, *p* = .00392 and *F. pruinosus*: χ^2^ = 4.4289, *df* = 1, *p* = .03533).

### 3.5 Association of common ant species in pitfalls with cactus density

**FIGURE 3:**
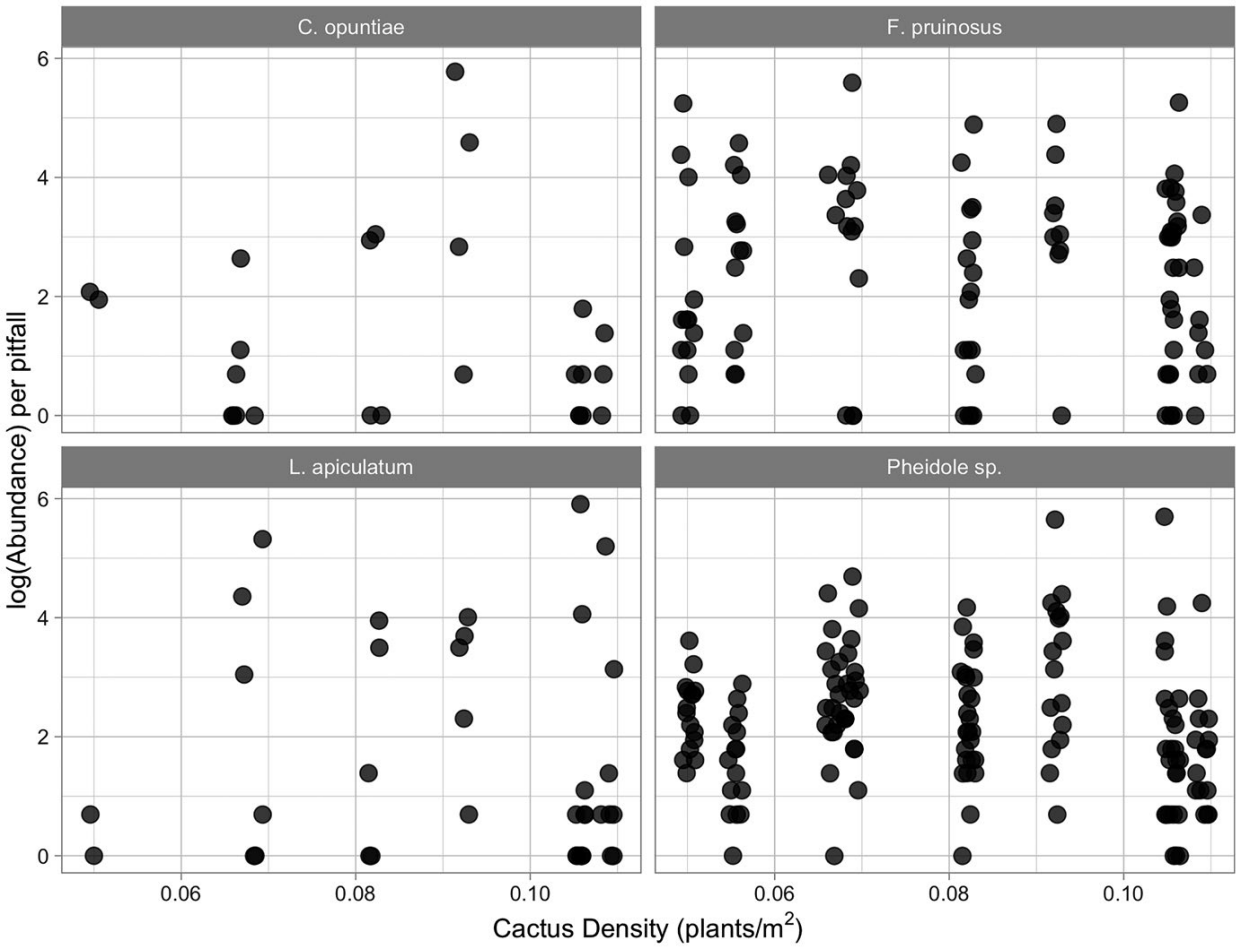
log(ant abundance) of each of the four most common ant species (different panels) across cactus density. Points represent log(abundance) from pitfall traps that had >0 ants for the given ant species

Results from the pitfall trapping study show the occurrence of the main ant partner *L. apiculatum* in pitfall traps was positively associated with increasing cactus density (Figure 2; χ^2^ = 11.462, *df* = 1, *p* = 0.0007). Specifically, the probability of *L. apiculatum* occurrence in the pitfall traps increased from 8% to 41% from low to high cactus density. Occurrence of the other two partner ant species was not significantly related to cactus density (Figure 2; *C. opuntiae*: χ^2^ = 0.9623, *df* = 1, *p* = .3266 and *F. pruinosus*: χ^2^ = 0.0067, *df* = 1, *p* = .9347). *Pheidole* sp. 1, the non‐partner species, showed a negative association in its occurrence in pitfall traps with increasing cactus density (χ^2^ = 4.7656, *df* = 1, *p* = .02903); however, this negative association was modest, as *Pheidole* occurred in 88.68% of all pitfall traps. Abundance per pitfall trap of these four ant species was not significantly associated with cactus density (Figure 3; null model vs. model with cactus density: *L. apiculatum* (χ^2^ = 0.0989, *df* = 1, *p* = .7531), *C. opuntiae* (χ^2^ = 0.1575, *df* = 1, *p* = .6914), *F. pruinosus* (χ^2^ = 0.3786, *df* = 1, *p* = .5383), and *Pheidole* sp. 1 (χ^2^ = 0.4193, *df* = 1, *p* = .5173)).

### 3.6 Association of common ant species in pitfalls with the dominant ant partner

The association of the occurrence and abundance of the three common ant species with those of *L. apiculatum* mirrored the results detected for cactus density. Only the occurrence of *Pheidole* sp. 1 in the pitfall traps was negatively associated with increasing occurrence of *L. apiculatum* (Figure S2; *C. opuntiae*: χ^2^ = 0.1956, *df* = 1, *p* = .658, *F. pruinosus*: χ^2^ = 0.3477, *df* = 1, *p* = .5554, and *Pheidole* sp. 1: χ^2^ = 4.104, *df* = 1, *p* = .04279). Abundance per pitfall trap of these three ant species was not significantly associated with abundance of *L. apiculatum* (Figure S3; null model vs. model with *L. apiculatum* abundance: *C. opuntiae* (χ^2^ = .2064, *df* = 1, *p* = .6496), *F. pruinosus* (χ^2^ = .1687, *df* = 1, *p* = .6812), and *Pheidole* sp. 1 (χ^2^ = 2.7825, *df* = 1, *p* = .0953)).

### 3.7 Association of ant community diversity and composition with cactus density

**FIGURE 4:**
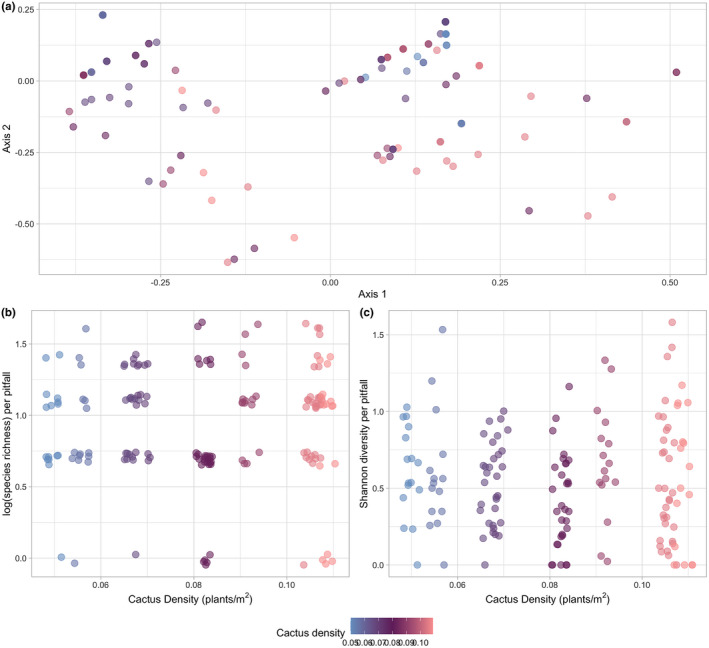
(a) PCoA of ant community composition with the Jaccard dissimilarity matrix. (b) Log(ant species richness) per pitfall trap across the cactus density gradient. (c) Shannon diversity (evenness) of the ant community within pitfall traps across the cactus density gradient. Points represent composition or diversity metric within a pitfall trap, and colors represent cactus density

Our results fail to reject the null hypothesis of no change in the ant community diversity or community composition in response to cactus density. Specifically, cactus density did not improve the model fits for log(species richness) (Figure 4b; pitfall: χ^2^ = 0.751, *df* = 1, *p* = .386, Figure 4b; plot: *df* = 1, *p* = .4837) nor Shannon diversity (Figure 4c; pitfall: χ^2^ = 0.0247, *df* = 1, *p* = .8752, Figure 4c; plot: *df* = 1, *p* = .8917) for communities at the pitfall or plot level, respectively. Additionally, community composition based on occurrence (Figure 4a; Jaccard) and abundance (Figure S4; Bray–Curtis) within pitfall traps was not significantly different across the cactus density gradient (PERMANOVA: Jaccard, *F* = 4.0611, *df* = 1, *R* ^2^ = .02537, *p* = 1; Bray–Curtis, *F* = 4.3018, *df* = 1, *R* ^2^ = .027, *p* = 1). Community composition pooled across pitfall traps within plots was not significantly different across cactus density (Figure S4; PERMANOVA: Jaccard, *F* = 1.0442, *df* = 1, *R* ^2^ = .11545, *p* = .429, Figure S6; Bray–Curtis, *F* = 1.7793, *df* = 1, *R* ^2^ = .18195, *p* = .144).

## 4 DISCUSSION

Since mutualisms are embedded within networks of interactions involving many non‐partner species, understanding the broader community‐level effects of mutualism is a key ecological goal. By tracking ant partners of an EFN‐producing cactus through time and space, as well as characterizing the effects of this partnership on the larger ant community, we addressed the hypothesis that mutualism is associated with increased ant partner occurrence and abundance and has cascading effects on the diversity and composition of the broader ant community. Our results partially support this hypothesis. We found that a single ant species (*L. apiculatum*) was the dominant partner across the 16‐year monitoring period. *L. apiculatum* partnership with cacti was positively associated with cactus density, and this relationship was also reflected with a positive correlation of occurrence in the environment (pitfall traps) with increasing cactus density. However, despite the consistency of the *L. apiculatum* partnership with the cacti through time, the dominance of this partnership at high cactus density, and the positive association of this partner ant species with increasing cactus density in the environment, broader consequences of this mutualism appear limited. The occurrence of one non‐partner ant species was negatively associated with cactus density, and this response was consistent with high *L. apiculatum* occurrence within pitfall traps. This may be indicative of the competitive effects of *L. apiculatum*. However, we found no evidence that ant community diversity and composition were affected by increasing densities of cacti and their dominant ant partner.

Our findings of a positive association of *L. apiculatum* occurrence, both on cacti and in pitfalls, with increasing cactus density support the idea of positive feedback between mutualist partners. Previous work demonstrated that cacti gain fitness benefits from protection by *L. apiculatum*, more so than other partner species (Miller, 2007; Ohm & Miller, 2014), and our new results suggest a reciprocal benefit of cactus‐derived resources on this dominant partner. The mutual promotion of partners is often assumed but rarely demonstrated in ant–plant studies (Prior et al., 2015; Wagner & Fleur Nicklen, 2010). One previous study found increased EFN production is associated with the increased establishment of ant nests (Wagner & Fleur Nicklen, 2010), thereby increasing ant partner presence near these plants. While we did not track *L. apiculatum* nests in our study, it is possible that *L. apiculatum* nests are more common in the high cactus density plots. Previous work has identified *L. apiculatum* colonies as being polydomous, meaning a colony will have satellite nests scattered across the landscape (Toro et al., 2009), and laboratory studies with a different polydomous ant species found that these nests were associated with foraging and formed near food resources (Lanan et al., 2011). The polydomous characteristic of *L. apiculatum* nests may help explain our finding of a positive association of occurrence but not abundance with increasing cactus density. Colony size may be capped, possibly due to other limiting resources, but ant colonies may shift their location to dominate areas of high EFN.

While cascading effects of cactus–*L. apiculatum* mutualism on non‐partner species appear limited, there was putative evidence that other partner species lose access to EFN where cacti reach high density and *L. apiculatum* is most abundant, suggesting competition for plant partners within the plant‐tending ant guild. While both *C. opuntiae* and *F. pruinosus* become infrequent partners at high cactus density, their occurrences within the pitfall traps did not show corresponding patterns of decrease. The occurrences of these two species within the pitfall traps were consistent across the gradient of cactus density, and in the case of *F. pruinosus*, this species’ abundance was generally high. For example, at high cactus density *F. pruinosus* occurred in approximately 75% of pitfall traps but tended nearly 0% of cacti, whereas *L. apiculatum* occurred in 40% of pitfalls but tended nearly 100% of plants. Taken together, our spatial sampling and long‐term data indicate that *L. apiculatum* is the dominant partner and that the shift in partner identities across space may be a result of competitive displacement from the cacti. These findings expand on previous research in this system that identified shifts in ant partner identity on individual cacti from *C. opuntiae* to *L. apiculatum* but not vice versa (Miller, 2007). Additionally, the fact that *C. opuntiae* and *F. pruinosus* did not decline in the environment even as they declined in their association with cacti at high cactus density suggests that these are opportunistic plant partners that are not strongly dependent on EFN. Indeed, in addition to EFN, *Crematogaster* spp. are broadly known to consume invertebrates (Palmer, 2003), forage on carrion (MacKay & Mackay, 2002), and nest in a variety of places, including in trees and logs and under rocks (MacKay & Mackay, 2002).

While the reason for low *L. apiculatum* partnership and occurrence in areas with low cactus density (where *C. opuntiae* was the most likely partner) is unclear, studies on different ant–plant symbioses have found that ant partner diversity can be maintained by habitat heterogeneity (Yu & Davidson, 1997) and trade‐offs among ant species, such as competition–colonization and dispersal–fecundity trade‐offs (Yu et al., 2001, 2004). As we did not detect a correlation between cactus density and ground cover composition, this may suggest that areas of high cactus density may be independent of areas with high‐value nesting and dietary resources. If this is indeed the case, the detected associations of ant species occurrence could be attributed to the shift in cactus density. However, belowground resources or other unmeasured habitat characteristics may also play contributing roles. What generates the variation in cactus density and what role the ant defenders play in maintaining this variation remain open questions. Further, while *L. apiculatum* has been previously described as a competitively dominant ant species (Andersen, 1997), little is known about its colonization, dispersal, and fecundity traits. Future studies characterizing these traits for *L. apiculatum* and the other partner ant species will be essential for discerning the landscape‐scale coexistence of multiple partner species.

Contrary to our expectation that positive feedback from ant–plant mutualism would result in spillover effects on the larger ant community, we found limited evidence for this. Specifically, we detected a modest negative association of *Pheidole* sp. 1 occurrence in areas with high cactus density and areas with high occurrence of *L. apiculatum*, but this ant species occurred in 88.68% of all pitfall traps, and when present, this species’ abundance did not correlate with the cactus density gradient. A previous study on ant behavioral dominance classified the *Pheidole* genus as moderately competitive, while it characterized the *Liometopum* genus as highly competitive (Andersen, 1997). Thus, this reduction from extremely high *Pheidole* occurrence to a moderate occurrence may reflect an avoidance response to areas with tree cholla tended by *Liometopum*. Otherwise, ant community diversity and composition appear to be unaffected. This is likely due to low niche overlap between the partner and non‐partner ant species. However, additional studies are necessary to determine which aspects of niche overlap contribute to structuring these observed patterns. As we did not detect a correlation of abundance of the four common ant species in our pitfall traps to cactus density, it is unsurprising that the community‐level abundance metrics (Shannon diversity and Bray–Curtis‐based community composition) did not respond to cactus density at either the pitfall‐ or plot‐level resolutions. However, abundance metrics should be interpreted cautiously, as the number of intercepted ants may not be representative of true colony abundance of a given species (Romero & Jaffe, 1989); pitfall traps may inadvertently intercept a worker trail or be biased due to differences in ant species’ movement behavior (Andersen, 1991), and thus disproportionately represent an ant species in the larger community. Our community‐level occurrence metrics (richness and presence/absence‐based community composition) provide a more conservative approach as this avoids the issue of inflated abundance. Yet, these metrics also did not identify a shift in species richness or composition across the cactus density gradient.

These findings of similar community composition across the cactus density gradient align with previous studies that found EFN‐producing plants did not strongly structure ant species composition (Belchior et al., 2016; Camarota et al., 2015), yet contrast with recent work that identified an increase in ant species richness and a shift in community composition with abundance of EFN‐producing trees (Ribeiro et al., 2018). Specifically, Ribeiro et al. (2018) characterized ant species richness on EFN‐bearing trees. Unlike tree cholla cacti at our study site, these trees were tended by multiple ant species at a time. Ribeiro et al. (2018) predicted and found that trees bearing EFN hosted higher ant species richness compared to trees without EFN, and ant species richness increased with the increase in EFN‐bearing tree frequency. Thus, this diffuse mutualism promoted ant species richness, while in our system, the exclusive monopolization of EFN was anticipated to reduce richness in the ant community. Our results suggest that despite a strong, positive association of a competitively dominant ant species’ occurrence—both on the cacti and in the environment—with increasing cactus density, ant community richness, evenness, and composition are unaffected by the suggested positive feedback between these mutualist partners. This lack of response may be due to dietary or nest site niche differences across partner and non‐partner ant species. While *L. apiculatum* is considered a broad generalist (Hoey‐Chamberlain et al., 2013), our long‐term data indicate that *L. apiculatum* also readily relies on EFN as a dietary resource. Thus, despite the positive association of *L. apiculatum* occurrence with areas of high cactus density, the dietary overlap across *L. apiculatum* and the co‐occurring ant species at these sites may be lessened due to the focus on EFN as a dietary resource. This may explain why we detected limited cascading effects on the larger ant community. Alternatively, it is possible that our sampling methods were too coarse to detect ant community responses, especially as many non‐partner ant species were often found in less than 10% of all pitfall traps. Further, while unlikely, it is possible that this desert environment may be a high‐resource setting where resource or nest site competition is not essential for structuring this ant community.

As with any observational study, our results should be interpreted in light of several limitations. While our ant partner surveys on the cacti were conducted over a 16‐year period, our community survey was a 24‐h snapshot in the spring. While we did not detect a community‐level response to increased *L. apiculatum* occurrence, it is possible that this outcome may differ across seasons. In the spring, cacti produce extrafloral nectar from both vegetative and reproductive parts. Previous work identified differences in the composition of extrafloral nectar from vegetative and reproductive parts, with EFN from reproductive segments having greater carbohydrate composition compared with nectar from stem segments Miller, 2014, and thus likely is a more valuable dietary resource to ants. It is possible that if reproductive parts reduce extrafloral nectar production later in the year (Robbins & Miller, 2009), this decrease in high‐quality resources may shift ant partner occurrence, which may cause changes in the ant community. To address this, studies could monitor ant partner identity on the cacti, as well as track the ant community through multiple seasons within a year. Additionally, as we did not experimentally manipulate cactus density, it is challenging to assign causality of our detected increase in *L. apiculatum* occurrence on the cacti and in the pitfall traps. Consideration should also be given to the roles of temperature, precipitation, nutrient, and resource availability contributing to the observed patterns. Experiments manipulating cactus density or exclusion of ants to the cacti and recording responses of ant species occurrence as partners and within the larger community through time would help clarify the role of cactus density in structuring partner identity and ant presence in the environment for this facultative mutualism.

To conclude, our results suggest that ant–plant mutualism can promote a dominant partner's occurrence in the landscape without affecting ant community richness, evenness, or composition and with only limited effects on individual non‐partner species. Instead of broad ant community spillover, our results suggest a more narrow consequence of ant–plant feedback whereby the dominant ant partner primarily displaced other partner species from EFN resources. Yet, these ant species and others are not displaced from the community, presumably due to niche partitioning. This study provides new evidence that even with apparent positive feedback between mutualist partners, their effects on community diversity and composition of co‐occurring species may be limited in scope. However, recent work has shown that cacti involved in this mutualism are predicted to increase in abundance under future climate warming (Czachura & Miller, 2020). Thus, future studies should address whether the result of limited spillover effects holds as these plants become more abundant, or whether at a certain cactus density threshold, the positive feedback between cactus density and *L. apiculatum* would result in strong spillover from this exclusive mutualism with negative consequences for ant community diversity and composition.

## CONFLICT OF INTEREST

The authors declare no conflicts of interest.

## AUTHOR CONTRIBUTIONS

**Marion L. Donald:** Conceptualization (equal); data curation (equal); formal analysis (lead); visualization (lead); writing—original draft (lead); writing—review and editing (equal). **Tom E. X. Miller:** Conceptualization (equal); data curation (equal); formal analysis (supporting); funding acquisition (lead); methodology (lead); project administration (lead); visualization (supporting); writing—original draft (supporting); writing—review and editing (equal).

## Supporting information

Appendix S1

## Data Availability

The long‐term cactus data are available from Miller 2020 (https://doi.org/10.6073/pasta/dd06df3f950afe4a4642306182237d13) on the EDI Data Portal. Pitfall community, ground cover, and cactus data from the additional plots are available in the Dryad repository (Donald and Miller 2021, https://doi.org/10.5061/dryad.gf1vhhmrb).

## References

[ece38524-bib-0001] Alonso, L. E. (1998). Spatial and temporal variation in the ant occupants of a facultative ant‐plant. Biotropica, 30(2), 201–213.

[ece38524-bib-0002] Andersen, A. (1991). Sampling communities of ground‐foraging ants: Pitfall catches compared with quadrat counts in an Australian tropical savanna. Australian Journal of Ecology, 16(3), 273–279. 10.1111/j.1442-9993.1991.tb01054.x

[ece38524-bib-0003] Andersen, A. N. (1997). Functional groups and patterns of organization in North American ant communities: A comparison with Australia. Journal of Biogeography, 24(4), 433–460. 10.1111/j.1365-2699.1997.00137.x

[ece38524-bib-0004] Aranda‐Rickert, A. , Diez, P. , & Marazzi, B. (2014). Extrafloral nectar fuels ant life in deserts. AoB PLANTS, 6, plu068. 10.1093/aobpla/plu068 25381258 PMC4262941

[ece38524-bib-0005] Báez, S. , Donoso, D. A. , Queenborough, S. A. , Jaramillo, L. , Valencia, R. , & Dangles, O. (2016). Ant mutualism increases long‐term growth and survival of a common amazonian tree. The American Naturalist, 188(5), 567–575. 10.1086/688401 27788348

[ece38524-bib-0006] Baker, H. G. , Opler, P. A. , & Baker, I. (1978). A comparison of the amino acid complements of oral and extrafloral nectars. Botanical Gazette, 139(3), 322–332.

[ece38524-bib-0007] Bates, D. , Mächler, M. , Bolker, B. , & Walker, S. (2015). Fitting linear mixed‐effects models using lme4g. Journal of Statistical Software, 67(1), 1–48.

[ece38524-bib-0008] Belchior, C. , Sendoya, S. F. , & Del‐Claro, K. (2016). Temporal variation in the abundance and richness of foliage‐dwelling ants mediated by extrafloral nectar. PLoS One, 11(7), e0158283. 27438722 10.1371/journal.pone.0158283 PMC4954677

[ece38524-bib-0009] Benson, L. (1982). The cacti of the United States and Canada (pp. 1044). Stanford University Press.

[ece38524-bib-0010] Blüthgen, N. , Stork, N. E. , & Fiedler, K. (2004). Bottom‐up control and co‐occurrence in complex communities: Honeydew and nectar determine a rainforest ant mosaic. Oikos, 106(2), 344–358. 10.1111/j.0030-1299.2004.12687.x

[ece38524-bib-0011] Bronstein, J. L. (1994). Conditional outcomes in mutualistic interactions. Trends in Ecology and Evolution, 9(6), 214–217. 10.1016/0169-5347(94)90246-1 21236825

[ece38524-bib-0012] Bronstein, J. L. , Alarcón, R. , & Geber, M. (2006). The evolution of plant–insect mutualisms. New Phytologist, 172(3), 412–428 10.1111/j.1469-8137.2006.01864.x 17083673

[ece38524-bib-0013] Byk, J. , & Del‐Claro, K. (2011). Ant‐plant interaction in the Neotropical savanna: Direct beneficial effects of extrafloral nectar on ant colony fitness. Population Ecology, 53(2), 327–332. 10.1007/s10144-010-0240-7

[ece38524-bib-0014] Camarota, F. , Powell, S. , Vasconcelos, H. L. , Priest, G. , & Marquis, R. J. (2015). Extrafloral nectaries have a limited effect on the structure of arboreal ant communities in a neotropical savanna. Ecology, 96(1), 231–240. 10.1890/14-0264.1 26236908

[ece38524-bib-0015] Chamberlain, S. A. , & Holland, J. N. (2009). Quantitative synthesis of context dependency in ant‐plant protection mutualisms. Ecology, 90(9), 2384–2392. 10.1890/08-1490.1 19769117

[ece38524-bib-0016] Corona, C. V. , del Carmen, M. , & Corona‐Vargas, R.‐M. (2007). *Liometopum apiculatum* (Formicidae: Dolichoderinae) y su relaci_on trofobio_tica con hemfn'nigptera Sternorrhyncha en Tlaxco, Tlaxcala, México. Acta Zoológica Mexicana, 23(2), 31–42.

[ece38524-bib-0017] Czachura, K. , & Miller, T. E. X. (2020). Demographic back‐casting reveals that subtle dimensions of climate change have strong effects on population viability. Journal of Ecology, 108(6), 2557–2570. 10.1111/1365-2745.13471

[ece38524-bib-0018] Davidson, D. W. (1997). The role of resource imbalances in the evolutionary ecology of tropical arboreal ants. Biological Journal of the Linnean Society, 61(2), 153–181. 10.1111/j.1095-8312.1997.tb01785.x

[ece38524-bib-0019] de Conconi, J. R. E. , Loaeza, R. M. , Aguilar, J. C. , & Rosas, G. S. (1983). Quelques données sur la biologie des fourmis *Liometopum* (Dolichoderinae) du Mexique et en particulier sur leurs rapports avec les homoptères. In P. Jaisson (Ed.), Social insects in the tropics (vol. 2, pp. 125–130). Université Paris‐Nord.

[ece38524-bib-0020] Fisher, B. L. , & Cover, S. P. (2007). Ants of North America: A guide to the genera. University of California Press.

[ece38524-bib-0021] Fraser, J. , & Pieper, R. (1972). Southwestern association of naturalists growth characteristics of Opuntia imbricata [Haw.] DC. in New Mexico. The Southwestern Naturalist, 17(3), 229–237. 10.2307/3670170

[ece38524-bib-0022] Frederickson, M. E. , Greene, M. J. , & Gordon, D. M. (2005). Ecology: ‘Devil's gardens’ bedevilled by ants. Nature, 437(7058), 495–496. 16177778 10.1038/437495a

[ece38524-bib-0023] Goslee, S. C. , & Urban, D. L. (2007). The ecodist package for dissimilarity‐based analysis of ecological data. Journal of Statistical Software, 22(7), 1–19.

[ece38524-bib-0024] Hartig, F. (2021). DHARMa: Residual diagnostics for hierarchical (multi‐level / mixed) regression models.

[ece38524-bib-0025] Hoey‐Chamberlain, R. , Rust, M. K. , & Klotz, J. H. (2013). A review of the biology, ecology and behavior of velvety tree ants of North America. Sociobiology, 60(1), 1–10. 10.13102/sociobiology.v60i1.1-10

[ece38524-bib-0026] Hölldobler, B. , & Wilson, E. O. (1990). The ants. Harvard University Press.

[ece38524-bib-0027] Horvitz, C. C. , & Schemske, D. W. (1990). Spatiotemporal variation in insect mutualists of a neotropical herb. Ecology, 71(3), 1085–1097. 10.2307/1937377

[ece38524-bib-0028] Koptur, S. (1992). Extrafloral nectary‐mediated interactions between insects and plants. In E. Bernays (Ed.), Insect‐plant interaction (pp. 81–129). CRC Press.

[ece38524-bib-0029] Lanan, M. C. , & Bronstein, J. L. (2013). An ant's‐eye view of an ant‐plant protection mutualism. Oecologia, 172(3), 779–790. 10.1007/s00442-012-2528-0 23515612 PMC4070855

[ece38524-bib-0030] Lanan, M. C. , Dornhaus, A. , & Bronstein, J. L. (2011). The function of polydomy: The ant 510 Crematogaster torosa preferentially forms new nests near food sources and fortifies outstations. Behavioral Ecology and Sociobiology, 65(5), 959–968.

[ece38524-bib-0031] Levings, S. C. , & Franks, N. R. (1982). Patterns of nest dispersion in a tropical ground ant community. Ecology, 63(2), 238–344.

[ece38524-bib-0032] MacKay, W. P. , & Mackay, E. (2002). The ants of New Mexico (Hymenoptera: Formicidae). Edwin Mellen Press.

[ece38524-bib-0033] Mayer, V. E. , Frederickson, M. E. , McKey, D. , & Blatrix, R. (2014). Current issues in the evolutionary ecology of ant‐plant symbioses. New Phytologist, 202(3), 749–764. 10.1111/nph.12690 24444030

[ece38524-bib-0034] Miller, T. E. X. (2007). Does having multiple partners weaken the benefits of facultative mutualism? A test with cacti and cactus‐tending ants. Oikos, 116(3), 500–512. 10.1111/j.2007.0030-1299.15317.x

[ece38524-bib-0035] Miller, T. E. X. (2014). Plant size and reproductive state affect the quantity and quality of rewards to animal mutualists. Journal of Ecology, 102(2), 496–507. 10.1111/1365-2745.12210

[ece38524-bib-0036] Ness, J. H. , Morris, W. F. , & Bronstein, J. L. (2016). Integrating quality and quantity of mutualistic service to contrast ant species protecting Ferocactus wislizeni. Ecology, 87(4), 912–921. 10.1890/0012-9658(2006)87[912:iqaqom]2.0.co;2 16676535

[ece38524-bib-0037] Ohm, J. R. , & Miller, T. E. X. (2014). Balancing anti‐herbivore benefits and anti‐pollinator costs of defensive mutualists. Ecology, 95(10), 2924–2935. 10.1890/13-2309.1

[ece38524-bib-0038] Oksanen, J. , Blanchet, F. G. , Friendly, M. , Kindt, R. , Legendre, P. , McGlinn, D. , Minchin, P. R. , O'Hara, R. B. , Simpson, G. L. , Solymos, P. , Stevens, M. H. H. , Szoecs, E. , & Wagner, H. (2019). Package ‘vegan’: Community ecology package. Tech. Rep. 9, 1–297, https://cran.r‐project.org/web/packages/vegan/index.html

[ece38524-bib-0039] Oliveira, P. S. , Rico‐gray, V. , & Castillo‐Guevara, C. D. C. A. C. (1999). Interaction between ants, extrafloral nectaries and insect herbivores in Neotropical coastal sand dunes: Herbivore deterrence by visiting ants increases fruit set in Opuntia stricta (Cactaceae). Functional Ecology, 13(5), 623–631.

[ece38524-bib-0040] Paine, R. T. (1966). Food web complexity and species diversity. The American Naturalist, 100(910), 65–75. 10.1086/282400

[ece38524-bib-0041] Palmer, T. M. (2003). Spatial habitat heterogeneity influences competition and coexistence in an African acacia ant guild. Ecology, 84(11), 2843–2855.

[ece38524-bib-0042] Palmer, T. M. , Stanton, M. L. , & Young, T. P. (2003). Competition and coexistence: Exploring mechanisms that restrict and maintain diversity within mutualist guilds. The American Naturalist, 162(4), S63–S79. 10.1086/378682 14583858

[ece38524-bib-0043] Pringle, E. G. , & Gordon, D. M. (2013). Protection mutualisms and the community: Geographic variation in an ant‐plant symbiosis and the consequences for herbivores. Sociobiology, 60(3), 242–251. 10.13102/sociobiology.v60i3.242-251

[ece38524-bib-0044] Prior, K. M. , Robinson, J. M. , Meadley Dunphy, S. A. , & Frederickson, M. E. (2015). Mutualism between co‐introduced species facilitates invasion and alters plant community structure. Proceedings of the Royal Society B: Biological Sciences, 282(1800), 20142846. 10.1098/rspb.2014.2846 PMC4298215 25540283

[ece38524-bib-0045] R Core Team . (2020). R: A language and environment for statistical computing. R Foundation for Statistical Computing. http://www.R‐project.org

[ece38524-bib-0046] Ribeiro, L. F. , Solar, R. R. C. , Muscardi, D. C. , Schoereder, J. H. , & Andersen, A. N. (2018). Extrafloral nectar as a driver of arboreal ant communities at the site‐scale in Brazilian savanna. Austral Ecology, 43(6), 672–680. 10.1111/aec.12612

[ece38524-bib-0047] Robbins, M. , & Miller, T. E. X. (2009). Patterns of ant activity on opuntia stricta (Cactaceae), a native host‐plant of the invasive cactus moth, cactoblastis cactorum (Lepidoptera: Pyralidae). Florida Entomologist, 92(2), 391–393.

[ece38524-bib-0048] Romero, H. , & Jaffe, K. (1989). A comparison of methods for sampling ants (Hymenoptera, Formicidae) in Savannas. Biotropica, 21(4), 348–352. 10.2307/2388285

[ece38524-bib-0049] Rosumek, F. B. , Silveira, F. A. O. , de S. Neves, F. , de U. Barbosa, N. P. , Diniz, L. , Oki, Y. , Pezzini, F. , Fernandes, G. W. , & Cornelissen, T. (2009). Ants on plants: A meta‐analysis of the role of ants as plant biotic defenses. Oecologia, 160(3), 537–549. 10.1007/s00442-009-1309-x 19271242

[ece38524-bib-0050] Rudgers, J. A. , & Clay, K. (2008). An invasive plant‐fungal mutualism reduces arthropod diversity. Ecology Letters, 11(8), 831–840. 10.1111/j.1461-0248.2008.01201.x 18479455

[ece38524-bib-0051] Rudgers, J. A. , Savage, A. M. , & Rúa, M. A. (2010). Geographic variation in a facultative mutualism: Consequences for local arthropod composition and diversity. Oecologia, 163(4), 985–996. 10.1007/s00442-010-1584-6 20198388

[ece38524-bib-0052] Stachowicz, J. J. (2001). The structure of ecological communities. BioScience, 51(3), 235–246.

[ece38524-bib-0053] Styrsky, J. D. , & Eubanks, M. D. (2007). Ecological consequences of interactions between ants and honeydew‐producing insects. Proceedings of the Royal Society B: Biological Sciences, 274(1607), 151–164. 10.1098/rspb.2006.3701 PMC1685857 17148245

[ece38524-bib-0054] Toby Kiers, E. , Palmer, T. M. , Ives, A. R. , Bruno, J. F. , & Bronstein, J. L. (2010). Mutualisms in a changing world: An evolutionary perspective. Ecology Letters, 13(12), 1459–1474. 10.1111/j.1461-0248.2010.01538.x 20955506

[ece38524-bib-0055] Toro, D. , Israel, J. P. , & Mackay, W. (2009). Revision of the ant genus Liometopum (Hymenoptera: Formicidae). Sociobiology, 53(2), 299–319.

[ece38524-bib-0056] Trager, M. D. , Bhotika, S. , Hostetler, J. A. , Andrade, G. V. , Rodriguez‐Cabal, M. A. , McKeon, C. S. , Osenberg, C. W. , & Bolker, B. M. (2010). Benefits for plants in ant‐plant protective mutualisms: A meta‐analysis. PLoS One, 5(12), e14308. 10.1371/journal.pone.0014308 21203550 PMC3008678

[ece38524-bib-0057] Traveset, A. , & Richardson, D. M. (2014). Mutualistic interactions and biological invasions. Annual Review of Ecology, Evolution, and Systematics, 45(1), 89–113. 10.1146/annurev-ecolsys-120213-091857

[ece38524-bib-0058] Wagner, D. , & Fleur Nicklen, E. (2010). Ant nest location, soil nutrients and nutrient uptake by ant associated plants: Does extrafloral nectar attract ant nests and thereby enhance plant nutrition? Journal of Ecology, 98(3), 614–624.

[ece38524-bib-0059] Yu, D. W. , & Davidson, D. W. (1997). Experimental studies of species‐specificity in Cecropia‐ant relationships. Ecological Monographs, 67(3), 273–294. 10.2307/2963456

[ece38524-bib-0060] Yu, D. W. , Wilson, H. B. , Frederickson, M. E. , Palomino, W. , De la colina, R. , Edwards, D. P. , & Balareso, A. A. (2004). Experimental demonstration of species coexistence enabled by dispersal limitation. Journal of Animal Ecology, 73(6), 1102–1114. 10.1111/j.0021-8790.2004.00877.x

[ece38524-bib-0061] Yu, D. W. , Wilson, H. B. , & Pierce, N. E. (2001). An empirical model of species coexistence in a spatially structured environment. Ecology, 82(6), 1761–1771.

