# Supplementary material for: Does ant–plant mutualism have spillover effects on the non‐partner ant community?
Source: Ecol Evol. 2022 Jan 24;12(1):e8524. doi: 10.1002/ece3.8524 (PMC8796954; doi:10.1002/ece3.8524)
Supplement: Supplementary file 1 — Appendix S1 [file ECE3-12-e8524-s001.pdf]

Figures and Table for Supplement

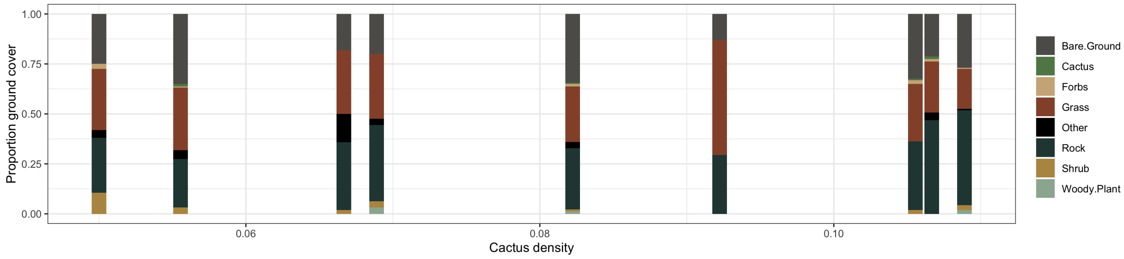

Figure S1: Proportion of each ground cover type across the range of cactus density. Ground cover types are shown in different colors. Note that two plots had a cactus density of 0.086 tree cholla m^{-2}. The ground cover for the duplicated cactus densities were pooled, which is why there are nine instead of 10 bars shown.

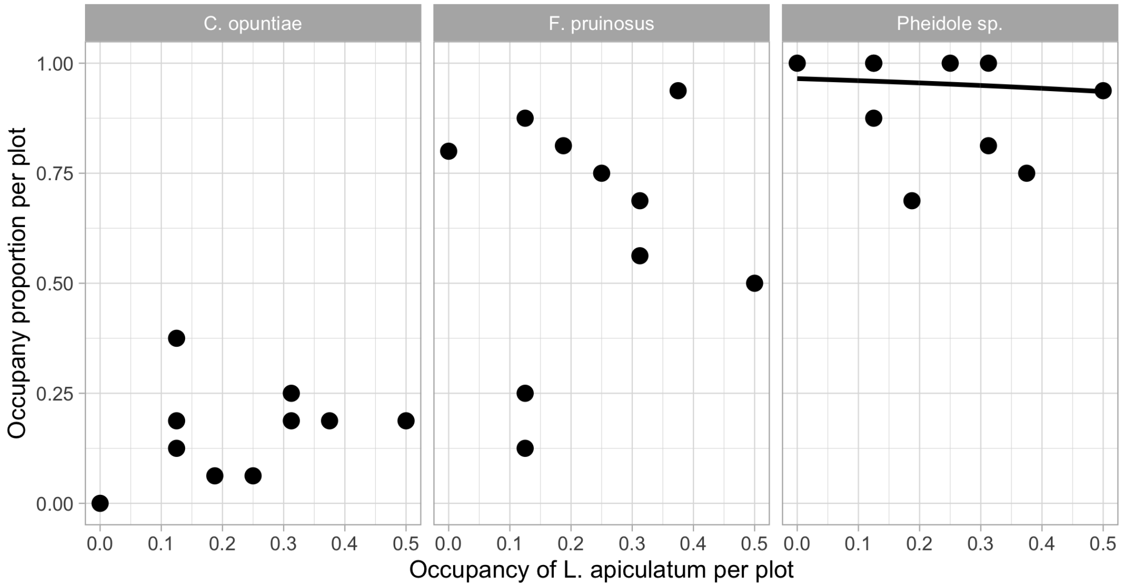

Figure S2: Occurrence of each of the three most common ant species (different panels) in response to the occurrence of *L. apiculatum*.

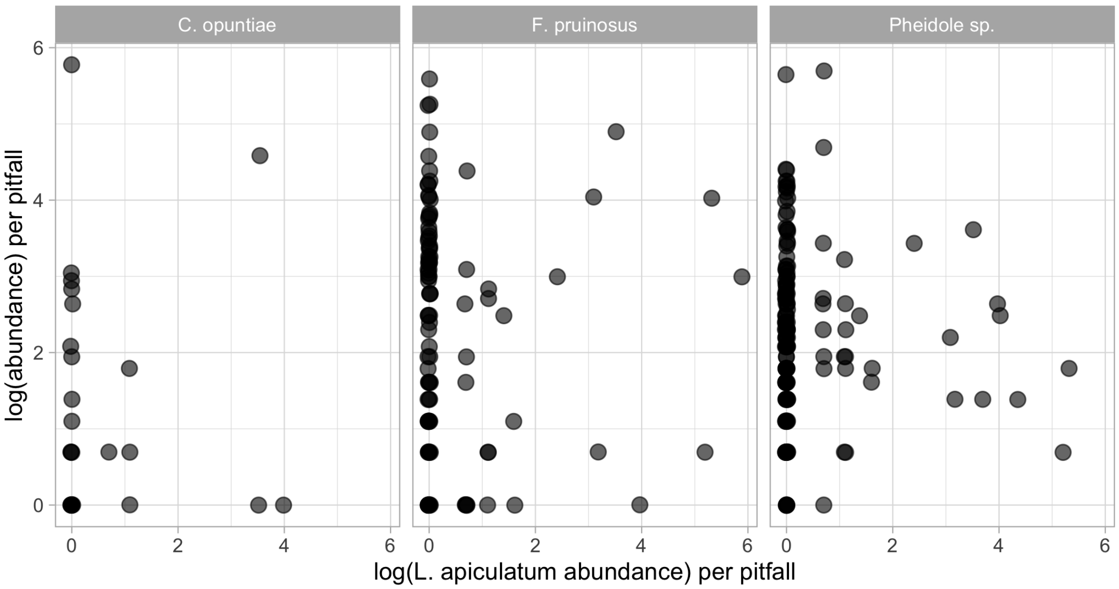

Figure S3: log(ant abundance) of each of the three most common ant species (different panels) in response to the abundance of *L. apiculatum*. Points represent log(abundances) from pitfall traps when the ant species was present.

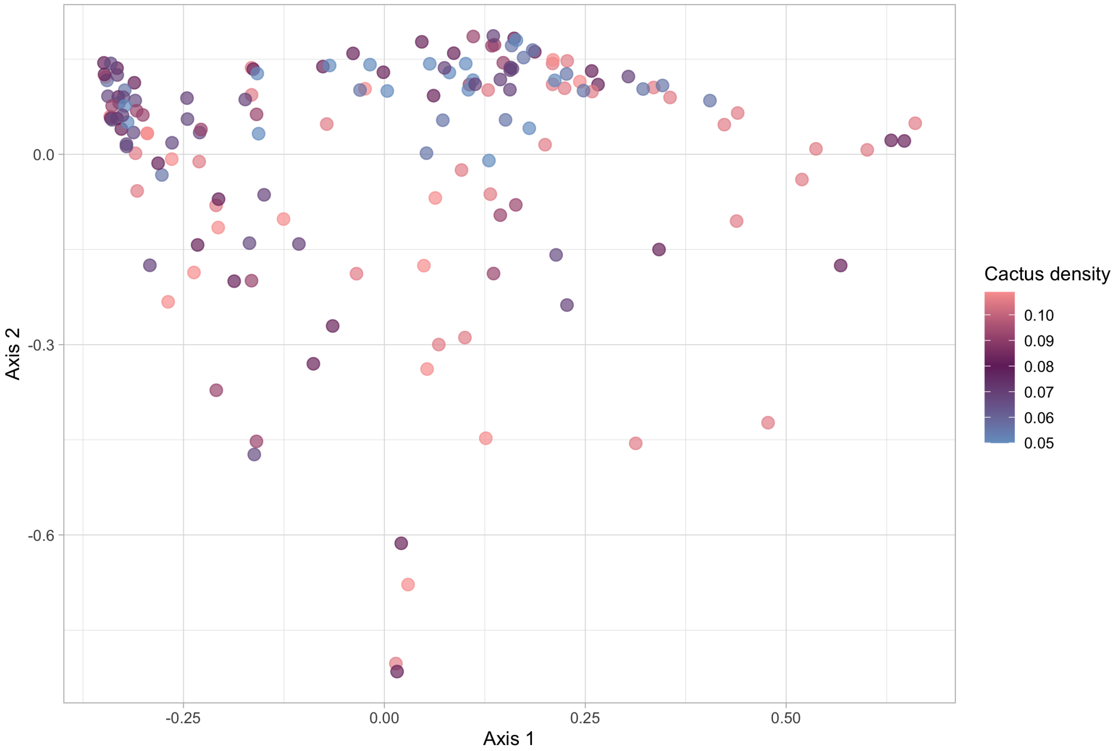

Figure S4: PCoA of ant community composition with the Bray-Curtis dissimilarity matrix. Points represent community composition within a pitfall trap and colors represent cactus density.

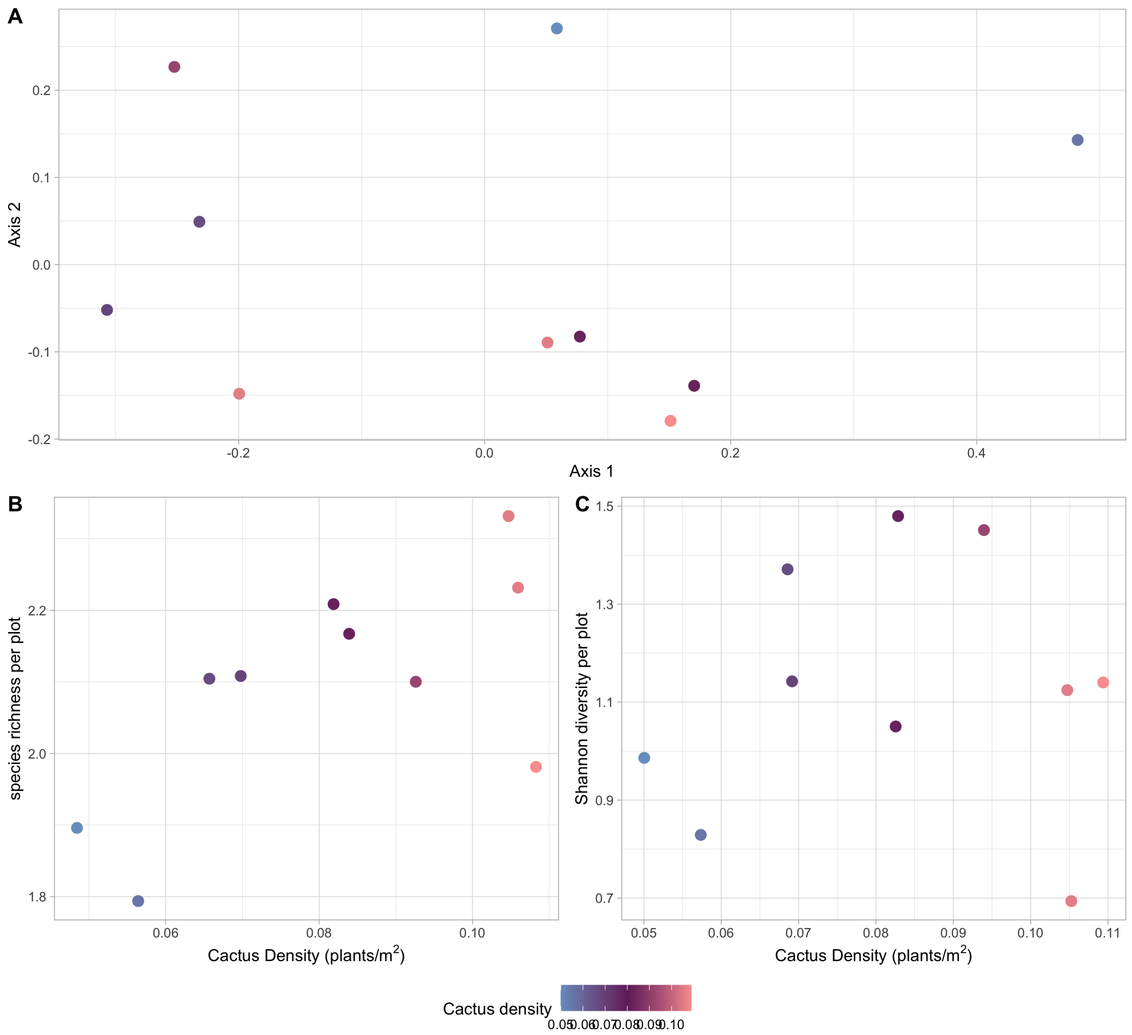

Figure S5: (A) PCoA of ant community composition at the plot level with the Jaccard dissimilarity matrix. (B) Ant species richness per plot across the cactus density gradient. (C) Shannon diversity (evenness) of the ant community per plot across the cactus density gradient. Points represent composition or diversity metric of all pitfall traps pooled within each plot and colors represent cactus density.

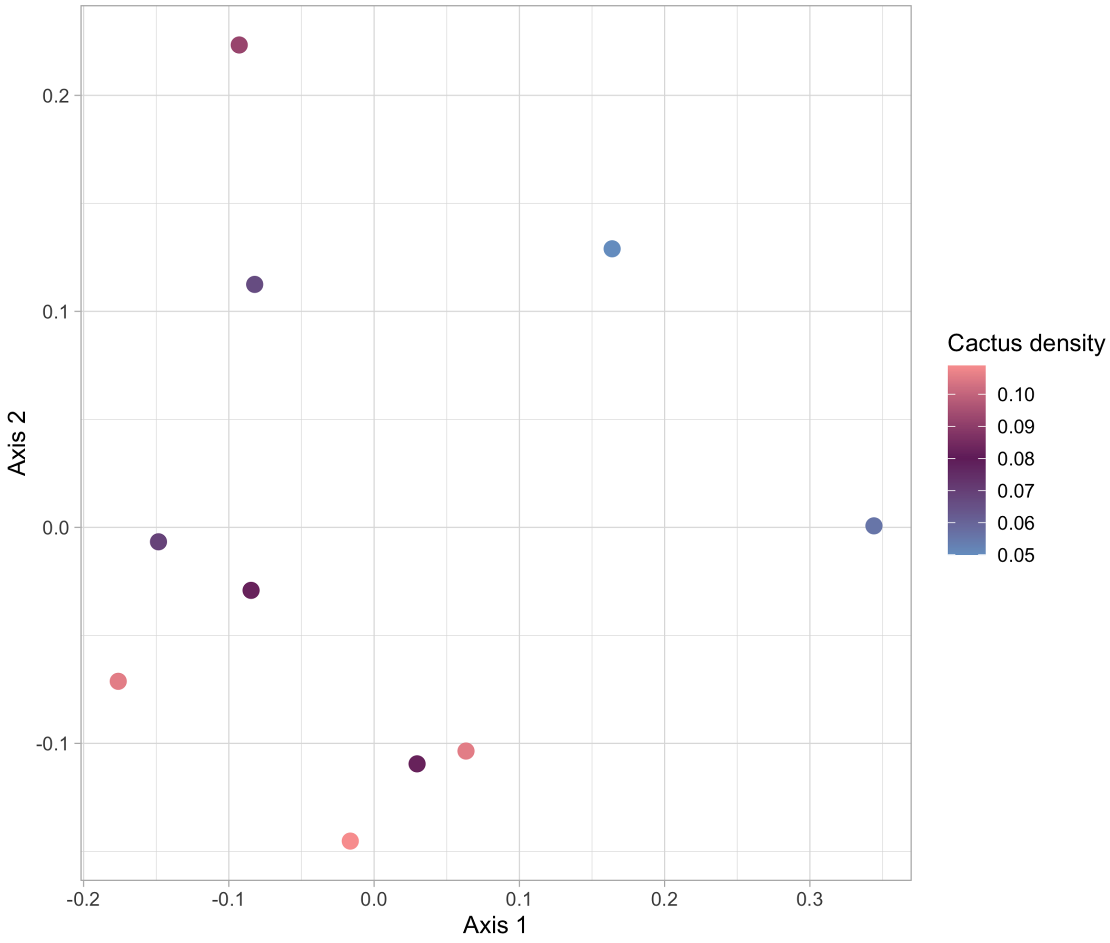

Figure S6: PCoA of ant community composition at the plot level with the Bray-Curtis dissimilarity matrix.

Table S1: Abundance and occurrence of each ant morphospecies

Morphospecies	Abundance (mean +/- SE)	Occurrence (%)
Brachymyrmex sp. 1	0.314 +/- 0.093	11.32
Camponotus sp. 1	0.113 +/- 0.032	8.81
Camponotus sp. 2	0.245 +/- 0.048	16.98
Camponotus sp. 3	0.057 +/- 0.022	4.40
Crematogaster opuntiae	3.390 +/- 2.127	16.35
Dorymyrmex sp. 1	0.987 +/- 0.411	9.43
Forelius pruinosus	17.145 +/- 2.914	62.89
Liometopum apiculatum	7.522 +/- 2.970	23.27
Pheidole sp. 1	17.799 +/- 2.970	88.68
Pogonomyrmex sp. 1	0.182 +/- 0.042	12.58
Pogonomyrmex sp. 2	0.050 +/- 0.021	3.77
Tetramorium sp. 1	0.220 +/- 0.079	10.69
Temnothorax sp. 1	0.006 +/- 0.006	0.63

Abundance is given as the mean +/- SE for each of the morphospecies. Occurrence is the proportion of total traps in which a morphospecies was found.
